# 4-(3-Fluoro-4-meth­oxy­phen­yl)-1-(4-meth­oxy­phen­yl)-5-(3,4,5-trimeth­oxy­phen­yl)-1*H*-imidazole

**DOI:** 10.1107/S160053681004465X

**Published:** 2010-11-06

**Authors:** Xiao-Meng Zhang, Cang Zhang, Wen-Ping Zhang, Xiao-Rong Liu

**Affiliations:** a Nanjing SanHome Pharmaceutical Institute, Nanjing 210038, People’s Republic of China

## Abstract

In the title mol­ecule, C_26_H_25_FN_2_O_5_, the fluoro­meth­oxy-, meth­oxy- and trimeth­oxy-substituted benzene rings form dihedral angles of 12.65 (2), 84.15 (2) and 55.67 (2)°, respectively, with the imidazole ring. The crystal structure is stabilized weak inter­molecular C—H⋯F and C—H⋯O hydrogen bonds.

## Related literature

For general background to the pharmacological activity of imidazole derivatives, see: Bellina *et al.* (2006 ▶, 2007 ▶); Cai *et al. *(2009 ▶). For background to synthetic methods for imidizoles, see: Bräuer *et al.* (2005 ▶), Wang *et al.* (2002 ▶).
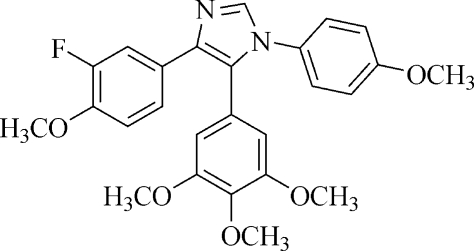


## Experimental

### 

#### Crystal data

C_26_H_25_FN_2_O_5_
*M* *_r_* = 464.48
Triclinic, 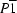

*a* = 9.795 (2) Å
*b* = 10.202 (2) Å
*c* = 13.008 (3) Å
α = 104.76 (3)°
β = 109.81 (3)°
γ = 91.45 (3)°
*V* = 1173.7 (4) Å^3^
*Z* = 2
Mo *K*α radiation
μ = 0.10 mm^−1^
*T* = 293 K
0.30 × 0.20 × 0.20 mm

#### Data collection

Enraf–Nonius CAD-4 diffractometer
Absorption correction: ψ scan (North *et al.*, 1968 ▶) *T*_min_ = 0.972, *T*_max_ = 0.981
4540 measured reflections
4269 independent reflections
2838 reflections with *I* > 2σ(*I*)
*R*_int_ = 0.024
3 standard reflections every 200 reflections  intensity decay: 1%

#### Refinement

*R*[*F* ^2^ > 2σ(*F* ^2^)] = 0.056
*wR*(*F* ^2^) = 0.178
*S* = 1.00
4269 reflections
308 parameters
H-atom parameters constrained
Δρ_max_ = 0.23 e Å^−3^
Δρ_min_ = −0.24 e Å^−3^

### 

Data collection: *CAD-4 EXPRESS* (Enraf–Nonius, 1994 ▶); cell refinement: *CAD-4 EXPRESS*; data reduction: *XCAD4* (Harms & Wocadlo, 1995 ▶); program(s) used to solve structure: *SHELXS97* (Sheldrick, 2008 ▶); program(s) used to refine structure: *SHELXL97* (Sheldrick, 2008 ▶); molecular graphics: *SHELXTL* (Sheldrick, 2008 ▶); software used to prepare material for publication: *SHELXL97*.

## Supplementary Material

Crystal structure: contains datablocks I, global. DOI: 10.1107/S160053681004465X/lh5129sup1.cif

Structure factors: contains datablocks I. DOI: 10.1107/S160053681004465X/lh5129Isup2.hkl

Additional supplementary materials:  crystallographic information; 3D view; checkCIF report

## Figures and Tables

**Table 1:** Hydrogen-bond geometry (Å, °)

*D*—H⋯*A*	*D*—H	H⋯*A*	*D*⋯*A*	*D*—H⋯*A*
C10—H10*A*⋯O3^i^	0.93	2.51	3.346 (4)	150
C26—H26*A*⋯F^ii^	0.96	2.52	3.326 (6)	142

Symmetry codes: (i) 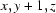; (ii) 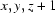.

**Fig. 1.**
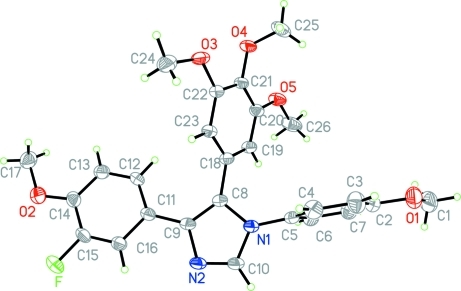
The molecular structure of (I), showing the atom-numbering scheme and displacement ellipsoids at the 30% probability level.

## supplementary crystallographic information

### Comment

Imidazole derivatives have been shown to exhibit interesting biological activities such as antimicrobial, anticryptococcal, inhibition of nitric oxide synthase, as inhibitors of p38 MAP kinase and cytotoxic activities (Bellina *et al.*, 2006; Bellina *et al.*, 2007; Cai *et al.*, 2009). Owing to these wide range of pharmacological and biological activities, the synthesis of imidazoles has become important (Bräuer *et al.*,2005; Wang *et al.*, 2002). Herein we present the crystal structure of the title compound (I).

The molecular structure of (I) is shown in Fig. 1. The dihedral angles that the fluoromethoxy, methoxy and trimethoxy-substituted, benzene rings form with the imidazole ring are 12.65 (2), 84.15 (2) and 55.67 (2)Å, respectively. The crystal structure is stabilized weak intermolecular C—H···F and C—H···N hydrogen bonds. .

### Experimental

To a solution of 4-methoxyaniline in (2.46 g, 20 mmol) in 5 ml/mmol methanol 3,4,5-trimethoxybenzaldehyde (3.92 g, 20 mmol) was added and stirred at room temperature. After 2 h the mixture was treated with 3,4,5-Trimethoxyphenyl(tosyl)methyl isocyanide (7.20 g, 20 mmol) and triethylamine (4.04 g, 40 mmol) and then heated under reflux for 8 h until the conversion of the starting material was complete. For the plate syntheses the reaction mixtures were heated on a sealed deep-well plate in a water bath at 323 K overnight. Aqueous workup was performed in the case of single reactions by adding ethyl acetate and washing the organic layer with 1 *M* aqueous HCl solution and aqueous NaCl solution. The organic layers were dried over anhydrous sodium sulfate and filtered. The filtrates were concentrated under vacuum, and purified using chromatographic methods described below. (Yield: 4.80 g, 51.9%; white solid; Mp. 429–430 K).

### Refinement

All the hydrogen atoms were placed in caculated positions with C—H = 0.93Å (aromatic) and 0.96Å (methyl) and *U*_iso_(H) = 1.2*U*_eq_(C) (aromatic C) and 1.5*U*_eq_(C) (methyl C).

### Crystal data

**Table:**

C_26_H_25_FN_2_O_5_	*Z* = 2
*M**_r_* = 464.48	*F*(000) = 488
Triclinic, *P*1	*D*_x_ = 1.314 Mg m^−^^3^
Hall symbol: -P 1	Mo *K*α radiation, λ = 0.71073 Å
*a* = 9.795 (2) Å	Cell parameters from 25 reflections
*b* = 10.202 (2) Å	θ = 9–13°
*c* = 13.008 (3) Å	µ = 0.10 mm^−^^1^
α = 104.76 (3)°	*T* = 293 K
β = 109.81 (3)°	Block, colourless
γ = 91.45 (3)°	0.30 × 0.20 × 0.20 mm
*V* = 1173.7 (4) Å^3^	

### Data collection

**Table:**

Enraf–Nonius CAD-4 diffractometer	2838 reflections with *I* > 2σ(*I*)
Radiation source: fine-focus sealed tube	*R*_int_ = 0.024
graphite	θ_max_ = 25.3°, θ_min_ = 1.7°
ω/–2θ scans	*h* = 0→11
Absorption correction: ψ scan (North *et al.*, 1968)	*k* = −12→12
*T*_min_ = 0.972, *T*_max_ = 0.981	*l* = −15→14
4540 measured reflections	3 standard reflections every 200 reflections
4269 independent reflections	intensity decay: 1%

### Refinement

**Table:**

Refinement on *F*^2^	Secondary atom site location: difference Fourier map
Least-squares matrix: full	Hydrogen site location: inferred from neighbouring sites
*R*[*F*^2^ > 2σ(*F*^2^)] = 0.056	H-atom parameters constrained
*wR*(*F*^2^) = 0.178	*w* = 1/[σ^2^(*F*_o_^2^) + (0.070*P*)^2^ + 1.1*P*] where *P* = (*F*_o_^2^ + 2*F*_c_^2^)/3
*S* = 1.00	(Δ/σ)_max_ < 0.001
4269 reflections	Δρ_max_ = 0.23 e Å^−^^3^
308 parameters	Δρ_min_ = −0.24 e Å^−^^3^
0 restraints	Extinction correction: *SHELXL97* (Sheldrick, 2008), Fc^*^=kFc[1+0.001xFc^2^λ^3^/sin(2θ)]^-1/4^
Primary atom site location: structure-invariant direct methods	Extinction coefficient: 0.063 (5)

### Special details

**Table:**

Geometry. All e.s.d.'s (except the e.s.d. in the dihedral angle between two l.s. planes) are estimated using the full covariance matrix. The cell e.s.d.'s are taken into account individually in the estimation of e.s.d.'s in distances, angles and torsion angles; correlations between e.s.d.'s in cell parameters are only used when they are defined by crystal symmetry. An approximate (isotropic) treatment of cell e.s.d.'s is used for estimating e.s.d.'s involving l.s. planes.
Refinement. Refinement of *F*^2^ against ALL reflections. The weighted *R*-factor *wR* and goodness of fit *S* are based on *F*^2^, conventional *R*-factors *R* are based on *F*, with *F* set to zero for negative *F*^2^. The threshold expression of *F*^2^ > σ(*F*^2^) is used only for calculating *R*-factors(gt) *etc*. and is not relevant to the choice of reflections for refinement. *R*-factors based on *F*^2^ are statistically about twice as large as those based on *F*, and *R*- factors based on ALL data will be even larger.

### Fractional atomic coordinates and isotropic or equivalent isotropic displacement parameters (Å^2^)

**Table:**

	*x*	*y*	*z*	*U*_iso_*/*U*_eq_	
F	−0.1298 (3)	0.7973 (2)	−0.06705 (17)	0.0787 (7)	
N1	0.2523 (3)	0.9865 (2)	0.5152 (2)	0.0456 (6)	
O1	0.5861 (3)	1.1432 (3)	0.9767 (2)	0.0814 (8)	
C1	0.5238 (6)	1.2059 (5)	1.0579 (3)	0.0915 (14)	
H1A	0.5975	1.2293	1.1327	0.137*	
H1B	0.4460	1.1437	1.0543	0.137*	
H1C	0.4859	1.2872	1.0417	0.137*	
N2	0.1490 (3)	1.0175 (2)	0.3441 (2)	0.0504 (7)	
O2	−0.2366 (3)	0.5452 (3)	−0.0943 (2)	0.0697 (7)	
C2	0.4976 (4)	1.1028 (3)	0.8645 (3)	0.0549 (8)	
O3	0.3163 (3)	0.3936 (2)	0.44874 (19)	0.0619 (7)	
C3	0.5629 (4)	1.0514 (4)	0.7881 (3)	0.0702 (11)	
H3A	0.6625	1.0436	0.8141	0.084*	
O4	0.2214 (3)	0.4035 (2)	0.61811 (19)	0.0558 (6)	
C4	0.4832 (4)	1.0104 (4)	0.6723 (3)	0.0607 (9)	
H4A	0.5287	0.9753	0.6207	0.073*	
O5	0.1123 (3)	0.6229 (2)	0.7079 (2)	0.0610 (7)	
C5	0.3357 (3)	1.0222 (3)	0.6343 (3)	0.0447 (7)	
C6	0.2704 (4)	1.0724 (3)	0.7116 (3)	0.0544 (8)	
H6A	0.1707	1.0795	0.6861	0.065*	
C7	0.3502 (4)	1.1125 (3)	0.8265 (3)	0.0575 (9)	
H7A	0.3046	1.1462	0.8782	0.069*	
C8	0.1876 (3)	0.8580 (3)	0.4414 (2)	0.0423 (7)	
C9	0.1239 (3)	0.8803 (3)	0.3364 (3)	0.0429 (7)	
C10	0.2242 (4)	1.0762 (3)	0.4503 (3)	0.0520 (8)	
H10A	0.2557	1.1694	0.4795	0.062*	
C11	0.0325 (3)	0.7870 (3)	0.2272 (3)	0.0432 (7)	
C12	−0.0271 (4)	0.6560 (3)	0.2128 (3)	0.0491 (8)	
H12A	−0.0059	0.6230	0.2762	0.059*	
C13	−0.1153 (4)	0.5736 (3)	0.1099 (3)	0.0552 (8)	
H13A	−0.1527	0.4864	0.1045	0.066*	
C14	−0.1503 (4)	0.6184 (3)	0.0124 (3)	0.0528 (8)	
C15	−0.0923 (4)	0.7503 (3)	0.0273 (3)	0.0516 (8)	
C16	−0.0043 (4)	0.8328 (3)	0.1282 (3)	0.0482 (8)	
H16A	0.0325	0.9199	0.1331	0.058*	
C17	−0.2849 (5)	0.4069 (4)	−0.1106 (3)	0.0742 (11)	
H17A	−0.3444	0.3664	−0.1890	0.111*	
H17B	−0.3412	0.4023	−0.0636	0.111*	
H17C	−0.2018	0.3582	−0.0901	0.111*	
C18	0.1942 (3)	0.7367 (3)	0.4859 (2)	0.0424 (7)	
C19	0.1424 (3)	0.7387 (3)	0.5724 (3)	0.0464 (7)	
H19A	0.0995	0.8133	0.5999	0.056*	
C20	0.1548 (3)	0.6278 (3)	0.6190 (3)	0.0455 (7)	
C21	0.2126 (3)	0.5149 (3)	0.5745 (3)	0.0444 (7)	
C22	0.2609 (3)	0.5107 (3)	0.4851 (3)	0.0457 (7)	
C23	0.2545 (3)	0.6233 (3)	0.4415 (3)	0.0451 (7)	
H23A	0.2901	0.6227	0.3836	0.054*	
C24	0.3521 (5)	0.3753 (4)	0.3498 (3)	0.0719 (11)	
H24A	0.3897	0.2894	0.3341	0.108*	
H24B	0.4248	0.4480	0.3616	0.108*	
H24C	0.2661	0.3761	0.2865	0.108*	
C25	0.3515 (5)	0.4118 (4)	0.7100 (3)	0.0786 (12)	
H25A	0.3507	0.3318	0.7359	0.118*	
H25B	0.3595	0.4915	0.7708	0.118*	
H25C	0.4334	0.4176	0.6859	0.118*	
C26	0.0553 (5)	0.7381 (4)	0.7569 (4)	0.0830 (13)	
H26A	0.0299	0.7219	0.8181	0.125*	
H26B	−0.0302	0.7540	0.7002	0.125*	
H26C	0.1276	0.8166	0.7858	0.125*	

### Atomic displacement parameters (Å^2^)

**Table:**

	*U*^11^	*U*^22^	*U*^33^	*U*^12^	*U*^13^	*U*^23^
F	0.1042 (17)	0.0838 (15)	0.0550 (12)	0.0114 (13)	0.0226 (12)	0.0389 (11)
N1	0.0594 (16)	0.0340 (13)	0.0464 (15)	0.0092 (11)	0.0185 (13)	0.0167 (11)
O1	0.0788 (19)	0.0852 (19)	0.0605 (17)	0.0057 (15)	0.0072 (15)	0.0113 (14)
C1	0.123 (4)	0.085 (3)	0.052 (2)	0.019 (3)	0.024 (3)	0.005 (2)
N2	0.0684 (18)	0.0358 (13)	0.0547 (17)	0.0137 (12)	0.0234 (14)	0.0232 (12)
O2	0.0814 (18)	0.0646 (16)	0.0522 (15)	0.0070 (13)	0.0110 (13)	0.0156 (12)
C2	0.061 (2)	0.0477 (19)	0.0484 (19)	0.0045 (16)	0.0119 (17)	0.0108 (15)
O3	0.1000 (19)	0.0350 (12)	0.0610 (15)	0.0220 (12)	0.0352 (14)	0.0209 (10)
C3	0.049 (2)	0.083 (3)	0.071 (3)	0.0134 (19)	0.0136 (19)	0.020 (2)
O4	0.0710 (15)	0.0387 (11)	0.0615 (14)	0.0044 (10)	0.0172 (12)	0.0295 (10)
C4	0.060 (2)	0.065 (2)	0.062 (2)	0.0126 (17)	0.0282 (19)	0.0176 (18)
O5	0.0809 (17)	0.0573 (14)	0.0681 (15)	0.0218 (12)	0.0422 (13)	0.0346 (12)
C5	0.0514 (19)	0.0316 (15)	0.0519 (18)	0.0052 (13)	0.0165 (15)	0.0155 (13)
C6	0.0480 (19)	0.054 (2)	0.061 (2)	0.0133 (15)	0.0212 (17)	0.0119 (16)
C7	0.065 (2)	0.054 (2)	0.054 (2)	0.0107 (17)	0.0245 (18)	0.0133 (16)
C8	0.0473 (17)	0.0326 (15)	0.0496 (18)	0.0093 (13)	0.0179 (14)	0.0149 (13)
C9	0.0481 (18)	0.0360 (15)	0.0533 (18)	0.0134 (13)	0.0222 (15)	0.0211 (14)
C10	0.071 (2)	0.0333 (16)	0.057 (2)	0.0118 (15)	0.0236 (18)	0.0201 (15)
C11	0.0494 (18)	0.0431 (17)	0.0466 (17)	0.0145 (14)	0.0221 (14)	0.0212 (14)
C12	0.059 (2)	0.0487 (18)	0.0434 (18)	0.0085 (15)	0.0148 (15)	0.0235 (15)
C13	0.058 (2)	0.0478 (19)	0.057 (2)	0.0069 (16)	0.0150 (17)	0.0185 (16)
C14	0.053 (2)	0.058 (2)	0.0455 (19)	0.0140 (16)	0.0131 (16)	0.0155 (16)
C15	0.066 (2)	0.059 (2)	0.0451 (19)	0.0219 (17)	0.0278 (17)	0.0281 (16)
C16	0.059 (2)	0.0453 (17)	0.0514 (19)	0.0146 (15)	0.0286 (16)	0.0203 (15)
C17	0.074 (3)	0.064 (2)	0.067 (2)	0.009 (2)	0.009 (2)	0.0104 (19)
C18	0.0471 (17)	0.0318 (15)	0.0469 (17)	0.0044 (13)	0.0122 (14)	0.0149 (13)
C19	0.0529 (19)	0.0378 (16)	0.0532 (18)	0.0113 (14)	0.0210 (15)	0.0177 (14)
C20	0.0471 (18)	0.0424 (17)	0.0512 (18)	0.0032 (14)	0.0172 (15)	0.0207 (14)
C21	0.0501 (18)	0.0338 (15)	0.0476 (17)	0.0015 (13)	0.0099 (14)	0.0194 (13)
C22	0.0550 (19)	0.0325 (15)	0.0460 (17)	0.0070 (13)	0.0115 (15)	0.0138 (13)
C23	0.0574 (19)	0.0376 (16)	0.0449 (17)	0.0096 (14)	0.0184 (15)	0.0187 (13)
C24	0.107 (3)	0.052 (2)	0.069 (2)	0.026 (2)	0.045 (2)	0.0175 (18)
C25	0.082 (3)	0.073 (3)	0.081 (3)	0.006 (2)	0.005 (2)	0.053 (2)
C26	0.108 (3)	0.092 (3)	0.094 (3)	0.049 (3)	0.067 (3)	0.056 (3)

### Geometric parameters (Å, °)

**Table:**

F—C15	1.369 (3)	C9—C11	1.459 (4)
N1—C10	1.369 (4)	C10—H10A	0.9300
N1—C8	1.395 (4)	C11—C12	1.387 (4)
N1—C5	1.429 (4)	C11—C16	1.416 (4)
O1—C2	1.367 (4)	C12—C13	1.360 (4)
O1—C1	1.418 (5)	C12—H12A	0.9300
C1—H1A	0.9600	C13—C14	1.394 (4)
C1—H1B	0.9600	C13—H13A	0.9300
C1—H1C	0.9600	C14—C15	1.387 (5)
N2—C10	1.295 (4)	C15—C16	1.345 (5)
N2—C9	1.388 (4)	C16—H16A	0.9300
O2—C14	1.358 (4)	C17—H17A	0.9600
O2—C17	1.417 (4)	C17—H17B	0.9600
C2—C3	1.361 (5)	C17—H17C	0.9600
C2—C7	1.374 (5)	C18—C19	1.379 (4)
O3—C22	1.365 (4)	C18—C23	1.395 (4)
O3—C24	1.415 (4)	C19—C20	1.403 (4)
C3—C4	1.385 (5)	C19—H19A	0.9300
C3—H3A	0.9300	C20—C21	1.377 (4)
O4—C21	1.386 (3)	C21—C22	1.389 (4)
O4—C25	1.404 (4)	C22—C23	1.398 (4)
C4—C5	1.380 (5)	C23—H23A	0.9300
C4—H4A	0.9300	C24—H24A	0.9600
O5—C20	1.366 (4)	C24—H24B	0.9600
O5—C26	1.411 (4)	C24—H24C	0.9600
C5—C6	1.367 (4)	C25—H25A	0.9600
C6—C7	1.376 (5)	C25—H25B	0.9600
C6—H6A	0.9300	C25—H25C	0.9600
C7—H7A	0.9300	C26—H26A	0.9600
C8—C9	1.377 (4)	C26—H26B	0.9600
C8—C18	1.488 (4)	C26—H26C	0.9600
			
C10—N1—C8	106.4 (2)	O2—C14—C15	117.8 (3)
C10—N1—C5	124.9 (3)	O2—C14—C13	125.8 (3)
C8—N1—C5	128.6 (2)	C15—C14—C13	116.4 (3)
C2—O1—C1	118.1 (3)	C16—C15—F	119.4 (3)
O1—C1—H1A	109.5	C16—C15—C14	123.6 (3)
O1—C1—H1B	109.5	F—C15—C14	117.0 (3)
H1A—C1—H1B	109.5	C15—C16—C11	120.1 (3)
O1—C1—H1C	109.5	C15—C16—H16A	120.0
H1A—C1—H1C	109.5	C11—C16—H16A	120.0
H1B—C1—H1C	109.5	O2—C17—H17A	109.5
C10—N2—C9	105.5 (2)	O2—C17—H17B	109.5
C14—O2—C17	117.4 (3)	H17A—C17—H17B	109.5
C3—C2—O1	116.5 (3)	O2—C17—H17C	109.5
C3—C2—C7	119.7 (3)	H17A—C17—H17C	109.5
O1—C2—C7	123.8 (3)	H17B—C17—H17C	109.5
C22—O3—C24	118.2 (2)	C19—C18—C23	120.5 (3)
C2—C3—C4	120.9 (3)	C19—C18—C8	119.3 (3)
C2—C3—H3A	119.5	C23—C18—C8	120.1 (3)
C4—C3—H3A	119.5	C18—C19—C20	119.9 (3)
C21—O4—C25	113.7 (2)	C18—C19—H19A	120.1
C5—C4—C3	119.3 (3)	C20—C19—H19A	120.1
C5—C4—H4A	120.4	O5—C20—C21	116.2 (3)
C3—C4—H4A	120.4	O5—C20—C19	124.1 (3)
C20—O5—C26	117.6 (3)	C21—C20—C19	119.7 (3)
C6—C5—C4	119.5 (3)	C20—C21—O4	120.0 (3)
C6—C5—N1	120.1 (3)	C20—C21—C22	120.6 (3)
C4—C5—N1	120.4 (3)	O4—C21—C22	119.4 (3)
C5—C6—C7	120.9 (3)	O3—C22—C21	115.0 (3)
C5—C6—H6A	119.6	O3—C22—C23	125.1 (3)
C7—C6—H6A	119.6	C21—C22—C23	119.8 (3)
C2—C7—C6	119.7 (3)	C18—C23—C22	119.3 (3)
C2—C7—H7A	120.1	C18—C23—H23A	120.3
C6—C7—H7A	120.1	C22—C23—H23A	120.3
C9—C8—N1	104.9 (2)	O3—C24—H24A	109.5
C9—C8—C18	134.8 (3)	O3—C24—H24B	109.5
N1—C8—C18	120.2 (3)	H24A—C24—H24B	109.5
C8—C9—N2	110.4 (3)	O3—C24—H24C	109.5
C8—C9—C11	130.6 (3)	H24A—C24—H24C	109.5
N2—C9—C11	118.9 (3)	H24B—C24—H24C	109.5
N2—C10—N1	112.8 (3)	O4—C25—H25A	109.5
N2—C10—H10A	123.6	O4—C25—H25B	109.5
N1—C10—H10A	123.6	H25A—C25—H25B	109.5
C12—C11—C16	116.3 (3)	O4—C25—H25C	109.5
C12—C11—C9	124.6 (3)	H25A—C25—H25C	109.5
C16—C11—C9	119.0 (3)	H25B—C25—H25C	109.5
C13—C12—C11	122.8 (3)	O5—C26—H26A	109.5
C13—C12—H12A	118.6	O5—C26—H26B	109.5
C11—C12—H12A	118.6	H26A—C26—H26B	109.5
C12—C13—C14	120.7 (3)	O5—C26—H26C	109.5
C12—C13—H13A	119.6	H26A—C26—H26C	109.5
C14—C13—H13A	119.6	H26B—C26—H26C	109.5
			
C1—O1—C2—C3	175.7 (4)	C17—O2—C14—C13	−6.2 (5)
C1—O1—C2—C7	−3.7 (5)	C12—C13—C14—O2	179.9 (3)
O1—C2—C3—C4	−178.6 (3)	C12—C13—C14—C15	−1.0 (5)
C7—C2—C3—C4	0.9 (6)	O2—C14—C15—C16	−179.3 (3)
C2—C3—C4—C5	0.1 (6)	C13—C14—C15—C16	1.4 (5)
C3—C4—C5—C6	−0.9 (5)	O2—C14—C15—F	1.4 (5)
C3—C4—C5—N1	177.2 (3)	C13—C14—C15—F	−177.9 (3)
C10—N1—C5—C6	83.9 (4)	F—C15—C16—C11	178.4 (3)
C8—N1—C5—C6	−97.9 (4)	C14—C15—C16—C11	−0.9 (5)
C10—N1—C5—C4	−94.1 (4)	C12—C11—C16—C15	−0.1 (4)
C8—N1—C5—C4	84.1 (4)	C9—C11—C16—C15	−177.8 (3)
C4—C5—C6—C7	0.7 (5)	C9—C8—C18—C19	−121.5 (4)
N1—C5—C6—C7	−177.3 (3)	N1—C8—C18—C19	55.6 (4)
C3—C2—C7—C6	−1.1 (5)	C9—C8—C18—C23	59.6 (5)
O1—C2—C7—C6	178.4 (3)	N1—C8—C18—C23	−123.3 (3)
C5—C6—C7—C2	0.3 (5)	C23—C18—C19—C20	2.1 (5)
C10—N1—C8—C9	−0.3 (3)	C8—C18—C19—C20	−176.8 (3)
C5—N1—C8—C9	−178.7 (3)	C26—O5—C20—C21	178.7 (3)
C10—N1—C8—C18	−178.2 (3)	C26—O5—C20—C19	−1.3 (5)
C5—N1—C8—C18	3.4 (5)	C18—C19—C20—O5	177.2 (3)
N1—C8—C9—N2	0.5 (3)	C18—C19—C20—C21	−2.9 (5)
C18—C8—C9—N2	177.9 (3)	O5—C20—C21—O4	1.7 (4)
N1—C8—C9—C11	−175.5 (3)	C19—C20—C21—O4	−178.2 (3)
C18—C8—C9—C11	2.0 (6)	O5—C20—C21—C22	−179.1 (3)
C10—N2—C9—C8	−0.5 (4)	C19—C20—C21—C22	1.0 (5)
C10—N2—C9—C11	176.0 (3)	C25—O4—C21—C20	−88.7 (4)
C9—N2—C10—N1	0.4 (4)	C25—O4—C21—C22	92.1 (4)
C8—N1—C10—N2	−0.1 (4)	C24—O3—C22—C21	172.9 (3)
C5—N1—C10—N2	178.5 (3)	C24—O3—C22—C23	−9.1 (5)
C8—C9—C11—C12	10.4 (5)	C20—C21—C22—O3	179.8 (3)
N2—C9—C11—C12	−165.3 (3)	O4—C21—C22—O3	−1.0 (4)
C8—C9—C11—C16	−172.2 (3)	C20—C21—C22—C23	1.7 (5)
N2—C9—C11—C16	12.1 (4)	O4—C21—C22—C23	−179.1 (3)
C16—C11—C12—C13	0.6 (5)	C19—C18—C23—C22	0.6 (4)
C9—C11—C12—C13	178.1 (3)	C8—C18—C23—C22	179.4 (3)
C11—C12—C13—C14	0.0 (5)	O3—C22—C23—C18	179.6 (3)
C17—O2—C14—C15	174.6 (3)	C21—C22—C23—C18	−2.5 (5)

### Hydrogen-bond geometry (Å, °)

**Table:**

*D*—H···*A*	*D*—H	H···*A*	*D*···*A*	*D*—H···*A*
C10—H10A···O3^i^	0.93	2.51	3.346 (4)	150
C26—H26A···F^ii^	0.96	2.52	3.326 (6)	142

Symmetry codes: (i) *x*, *y*+1, *z*; (ii) *x*, *y*, *z*+1.
